# H258R mutation in *KCNAB3* gene in a family with genetic epilepsy and febrile seizures plus

**DOI:** 10.1002/brb3.1859

**Published:** 2020-09-29

**Authors:** Jian Ding, Qin‐Fei Miao, Jing‐Wen Zhang, Yu‐Xiong Guo, Yu‐Xin Zhang, Qiong‐Xiang Zhai, Zhi‐Hong Chen

**Affiliations:** ^1^ Department of Pediatrics Affiliated Dongguan People's Hospital Southern Medical University Dongguan China; ^2^ Department of Pediatrics Guangdong Provincial People's Hospital Guangdong Academy of Medical Sciences, Guangdong Academy of Neuroscience Guangzhou China; ^3^ Southern Medical University Guangzhou China

**Keywords:** Chinese pedigree, genetic epilepsy with febrile seizures plus (GEFS+), patch‐clamp technique, potassium voltage‐gated channel subfamily A regulatory beta subunit 3 (KCNAB3), whole‐exome sequencing

## Abstract

**Purpose:**

The aim of this was to discover disease‐causing gene mutations linked to genetic epilepsy with febrile seizures plus (GEFS+) in a family in the Southern Chinese Han population. Of a three‐generation pedigree of 18 members in this family, 4 were affected with GEFS+.

**Method:**

Blood samples of 7 family members—3 affected and 4 unaffected individuals—were collected. Whole‐exome sequencing was performed to assess for genetic mutations in two of the affected individuals and two of the unaffected individuals.

**Results:**

Fourteen potentially consequential mutations were found in the two affected individuals and were validated with the Sanger sequencing method. Blood DNA tested in polymerase chain reaction with *KCNAB3* primers revealed that one novel missense mutation, c.773A>G (p.H258R) in the *KCNAB3* gene, which encoded the potassium voltage‐gated channel subfamily A regulatory β subunit 3 (*KCNAB3*), was shared by all three affected and one unaffected family member. However, this mutation did not appear in 300 unrelated control subjects. According to the bioinformatics tools SIFT and PROVEAN, p.H258R was thought to affect protein function. Functional verification showed that the *KCNAB3* mutation could accelerate the inactivation of potassium channels, thus inhibiting potassium current, increasing neuronal excitability, and promoting epileptic convulsion.

**Conclusions:**

These results reveal that mutations in the *KCNAB3* gene may be associated with GEFS+.

## Graphical abstract

A novel missense mutation in KCNAB3 was found in sufferers of a Chinese Han GEFS+ family using whole‐exome sequencing. Functional verification showed that KCNAB3 mutation could accelerate the inactivation of potassium channels, thus inhibiting potassium current, improving neuronal excitability, and promoting epileptic convulsion. It is the first report about mutation in KCNAB3 gene relating to GEFS+, and these findings may provide a new insight that mutations in the KCNAB3 gene may be the pathology of GEFS+.

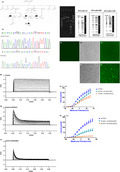

## 1 INTRODUCTION

Genetic epilepsy with febrile seizures plus (GEFS+) is an epilepsy syndrome with familial and phenotypic heterogeneity (Myers et al., 2017). Its phenotypic spectrum varies from mild classical febrile seizures to severe Dravet syndrome (DS) (Scheffer & Berkovic, 1997). GEFS+ is inherited through autosomal dominance (Mulley et al., 2013). Mutations in *SCN1A, SCN1B, SCN9A, STX1B,* and *GABRG2* have been reported to be linked to GEFS+ (ILAE Commission & on Classification & Terminology, 2014). *SCN1A* is the gene most frequently observed in affected patients, accounting for about 11% of large families with affected members (Marini et al., 2007).

However, these mutations exist in only a small percentage of families whose members have GEFS+; this may indicate that other genes contribute to this disorder. In this study, we evaluated members of a family in the South Chinese Han population in whom GEFS+ was diagnosed according to the typical disease phenotype, and we used whole‐exome sequencing to detect potential mutations that were responsible for the disease.

## 2 MATERIALS AND METHODS

### 2.1 Patients

**Figure 1:**
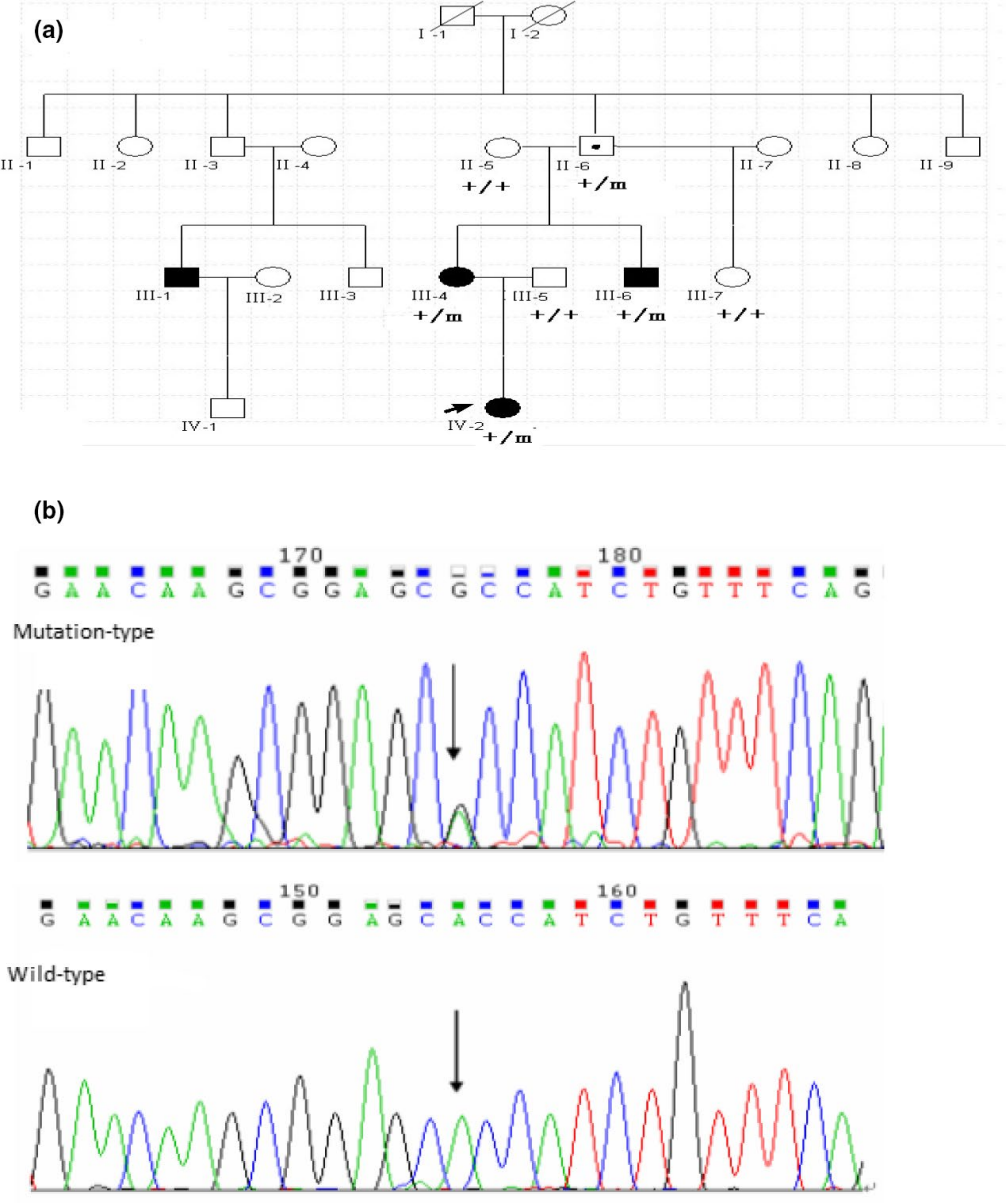
A Chinese GEFS+ pedigree with a mutation in the *KCNAB3* gene. (a) GEFS+ pedigree with c.773A>G mutation in the KCNAB3 gene. Filled symbols indicate affected individuals and clear symbols unaffected individuals; squares: males; circles: females; arrow represents the proband. Genotypes are indicated for each individual. “+” means wild type; “m” means mutant. (b) The c.773A>G mutation (↓) replaces H (Histidine) with R (Arginine)

Strictly in accordance with the 2017 International League Against Epilepsy diagnostic classification criteria for epilepsy and epilepsy syndrome, we recruited 118 patients with epilepsy or epilepsy syndrome from 23 families. The diagnosis was based on the clinical history, physical examination findings, electroencephalogram/ictal video electroencephalogram, video polysomnographic analysis, and cranial magnetic resonance imaging (MRI). In one of these families, a three‐generation pedigree from the Han population in southern China included 18 members, 4 of whom had a diagnosis of GEFS+ (Figure 1). Three affected and four unaffected members of this family took part in this study. The average age at onset was 1 year and 4 months and ranged from 10 months to 2 years. We also recruited a control group of 300 healthy volunteers from the same region and of the same nationality who did not have epilepsy or related diseases. All subjects and healthy controls received provided informed consent after learning about the nature of the study and possible results. The research program was approved by the Human Research Ethics Committee of Guangdong provincial people's hospital, according to the principles of the Helsinki Declaration.

### 2.2 Exome sequencing and analysis

The QIAamp DNA Blood Mini Kit (QIAGEN Inc., Hilden, Germany) was used to extract DNA from subjects' peripheral blood. Two affected and two unaffected individuals from the GEFS+ pedigree (II‐6, III‐4, III‐5, and IV‐2 in Figure 1) were selected for exome sequencing. BigDye^®^ Terminator v3.1 Cycle Sequencing Kit (Applied Biosystems Inc.) was used for exome capture. According to the manufacturer's instructions (BGI Inc.), we sequenced exon‐enriched DNA on an Illumina Genome Analyzer II platform (Illumina). The paired‐end sequences produced by massively parallel sequencing were 90 bp long, which is more than 125 times the average read length of such sequences and covers more than 98% of the target area, thereby conforming to quality standards.

Using Burrows–Wheeler Alignment software, we compared the raw data with the reference human genome (GRCh37). The potential pathogenicity variant was derived in step‐by‐step filtering. The filtering removed from analysis the common variants with minor allele frequency of 0.005 or less, according to the recommendations of the 1000 Genomes Project, the Exome Aggregation Consortium (ExAC), or the ExAC‐East Asian population; in addition, the disease‐related genes previously reported in the Human Genetic Mutation Database and in the Online Mendelian Inheritance in Man (OMIM) database were removed. The pathogenicity variants were predicted by the Protein Variation Effect Analyzer (PROVEAN) software tool (http://provean.jcvi.org/) and Sorting Intolerant From Tolerant (SIFT) software, version 5.2.2 (http://sift.jcvi.org/). PROVEAN helps predict whether amino acid changes affect protein function. It not only supports quick determination of protein variants from all living bodies but also provides high‐throughput analysis at the genomic and protein levels for human and mouse variants (Choi & Chan, 2015). SIFT helps predict the functional effect of an amino acid substitution on sequence homology and the physical natures of amino acids (Kumar et al., 2009).

### 2.3 Patch‐clamp experimental method

**Figure 2:**
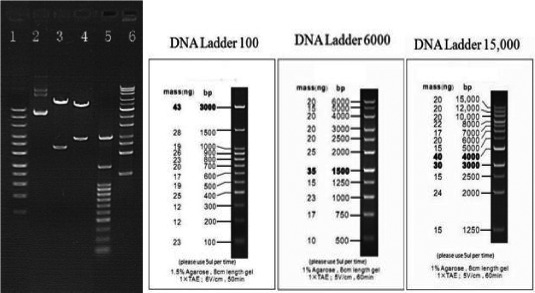
Plasmid digestion diagram. Lane1: Marker 6000; Lane2: Plasmid of pEZ‐KCNAB3 H258R‐Lv201; Lane3: Plasmid of pEZ‐KCNAB3 H258R‐Lv201 cut by BsrGI. There are two bands (~7459/2371bp); Lane4: Plasmid of pEZ‐KCNAB3 H258R‐Lv201 cut by AflII. There are two bands (~6870/2960bp); Lane5: Marker 3000; Lane6: Marker 15000

The coding regions of *KCNAB3* (NM_004732.3) and *KCNA1* (NM_000217) were obtained from the National Center for Biotechnology Information. Primers were designed according to the coding region, and the target fragments were acquired by polymerase chain reaction (PCR). The target fragments and the lentivirus vector plasmid (pEZ‐Lv201) were cut by restriction enzymes and linked to form the cloned recombinant, and then, the recombinant plasmids pEZ‐KCNAB3 Wt‐Lv201 and pEZ‐KCNA1 Wt‐Lv201 were obtained. Using point mutation‐specific PCR, we obtained the recombinant plasmid pEZ‐KCNAB3 H258R‐Lv201, with the primer‐designed center on the mutation site, pEZ‐KCNAB3 Wt‐Lv201, as the template. We performed plasmid transformation by using OmniMAX 2 T1 chemically competent cells. PCR was used to screen the recombinant plasmids, and the PCR products were detected by means of agarose gel electrophoresis. The plasmid containing the target fragment was identified with enzyme digestion (Figure 2) and verified by sequencing.

**Figure 3:**
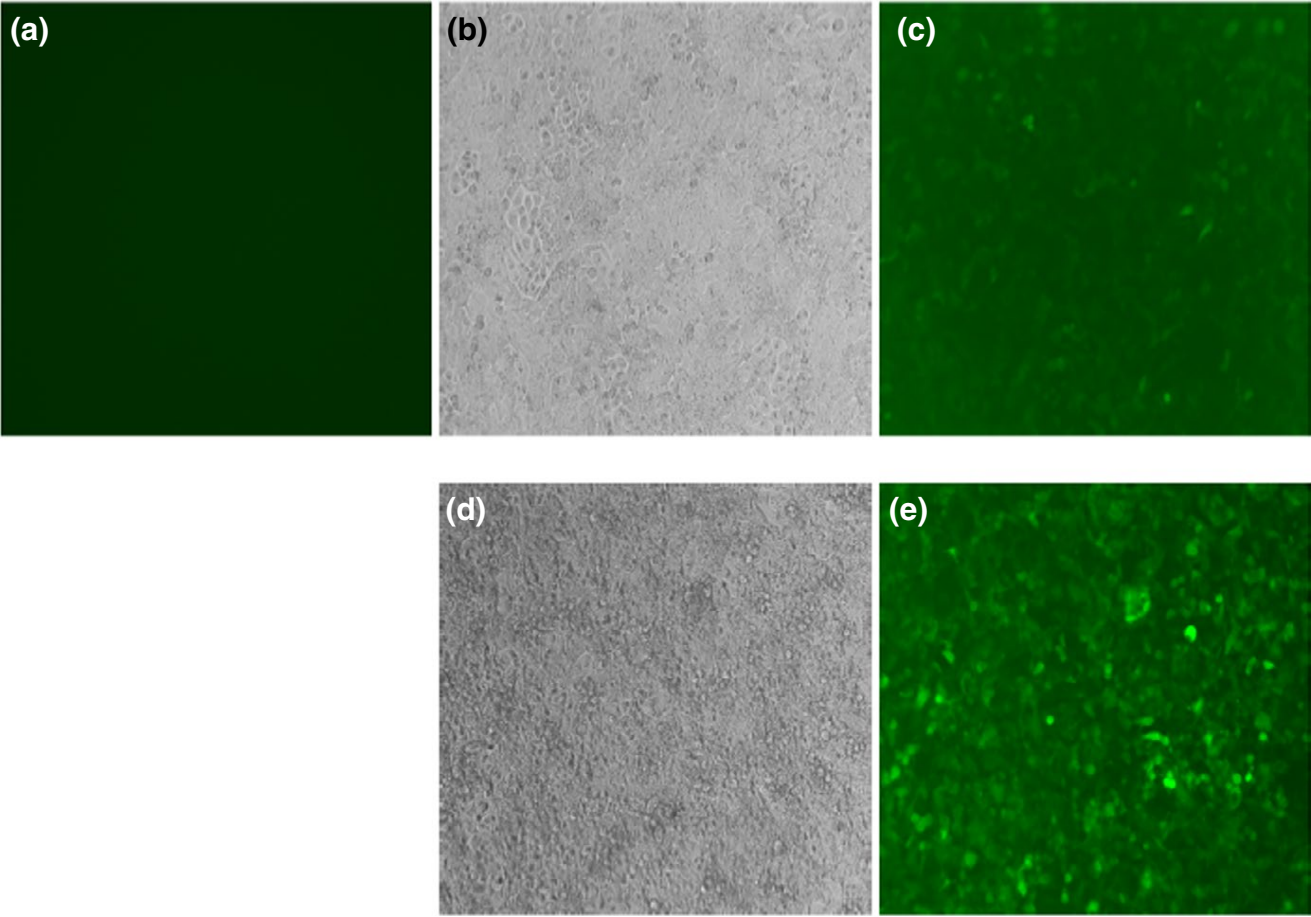
Fluorescent images of H1299 cells. (a) H1299 cells alone; (b) H1299 cells transduced with pEZ‐KCNAB3 H258R‐Lv201, phase contrast, 1 μl; (c) H1299 cells transduced with pEZ‐KCNAB3 H258R‐Lv201, 1 μl; (d) H1299 cells transduced with pEZ‐KCNAB3 H258R‐Lv201, phase contrast, 1 μl (drug selection with Puromycin). (e) H1299 cells transduced with pEZ‐KCNAB3 H258R‐Lv201, 1 μl (drug selection with Puromycin)

After verification, the lentivirus plasmid was packaged in 293Ta cells, and fluorescence detection was performed in H1299 cells (Figure 3). Human embryonic kidney cells (HEK293) were cultured in Dulbecco's modified Eagle medium, which contained 10% heat‐inactivated fetal bovine serum, in 5% CO_2_ and at 37°C until the logarithmic growth period; then, they were transfected with the calculated lentivirus plasmid. Reverse transcription PCR and fluorescence detection were performed after transfection. The transfected cells were then placed into a recording pool containing extracellular fluid (140 mmol of NaCl, 3.5 mmol of KCl, 1 mmol of MgCl_2_, 2 mmol of CaCl_2_, 10 mmol of glucose, 10 mmol of HEPES, and 1.25 mmol of NaH_2_PO_4_ at a pH of 7.4 with NaOH) and the corresponding intracellular fluid (5 mmol of NaCl, 140 mmol of potassium gluconate, 1 mmol of MgCl_2_, 0.1 mmol of CaCl_2_, 1 mmol of ethylene glycol tetraacetic acid, 10 mmol of HEPES, and 2 mmol of Mg adenosine triphosphate at a pH of 7.2 with KOH), which should be used together.

A microelectrode puller (P‐97; Sutter Instruments) was used to make recording electrodes by pulling the capillary micropipette (BF150‐86‐10; Sutter Instruments). Operating the microelectrode manipulator (MP‐285; Sutter Instruments) under an inverted microscope (IX71; Olympus, Tokyo, Japan) allowed the recording electrode to touch the cell, and with negative pressure suction, GΩ sealing was formed. After formation of the GΩ sealing, compensated quick capacitance kept the negative pressure, and the cell membrane was aspirated to form a whole cell recording mode. Then, compensated slow capacitance, recorded cell membrane capacitance, and series resistance were measured. No leakage compensation was observed. When the whole cell recording mode was stable, started to record current (current voltage characteristic [I‐V] curve) (*n* = 11). Multiple cells were independently evaluated repeatedly during recording. All electrophysiological experiments were performed at room temperature.

## 3 RESULTS

### 3.1 Description of the pedigree

The initial proband of this GEFS+ family was a 3‐year‐old girl, who was born at full term after a normal pregnancy and whose early psychomotor development was normal. Beginning at the age of 10 months, she suffered more than eight febrile seizures and one afebrile seizure. During every seizure, electroencephalography showed a generalized tonic‐clonic pattern that lasted several minutes. Neurological examination revealed no obvious abnormality, and brain MRI yielded unremarkable results. The interictal electroencephalogram showed bilateral symmetrical, multifocal, frequent sharp, and slow waves. The clinical phenotype was diagnosed as febrile seizures plus, and the patient responded well to sodium valproate treatment. Other affected members in this GEFS+ family also had febrile seizures. The unaffected individual carrying the mutation have a normal phenotype (Table 1).

**Table 1:** Main clinical characteristics of four patients of a GEFS+ pedigree in the Southern Chinese Han population

No.	Sex	Age at time of study (years)	Age at onset (years)	Clinical phenotype	Intellectual disability	Psychiatric disorder	Interictal EEG	MRI	Drug
III−1	M	15	2	FS	N	N	Single sharp wave	N	No
III−4	F	34	1	FS	N	N	Single sharp wave	N	No
III−6	M	32	2	FS	N	N	N	N	No
IV−2	F	3	10/12*	FS+	N	N	bilateral symmetrical multifocal frequent sharp and slow waves	N	sodium valproate

Note

F, female; M, male; N, normal; *, 10 months.

### 3.2 Exome sequencing

We found 31 gene variants in two affected members of the pedigree (Table 2). Sanger sequencing was used to confirm these variants; of the 31 variants, 17 turned out to false positive. The remaining 14 gene variants were used as the target gene for verification in 7 family members. Only 1 variant in the *KCNAB3* gene was found in all three affected individuals, this variant was also present in one unaffected individual; it meets the criteria of coisolation of phenotype and genotype. The other gene mutations did not confirm the criteria of phenotype and genotype coisolation after verification; thus, they were not considered to be pathogenic genes for GEFS+ (Table 3).

**Table 2:** Primers sequence and annealing temperature of mutant genes co‐exist in two affected members in pedigree

GeneName	5′−3′	3′−5′	annealed temperature (°C)
DNAH3	AGCATAGTTTTGGCTCTGGT	CTTTCCCTCAGGCCACTCAG	60
EARS2	CTCTGCTCCCCATCAGGTAT	CGTACCTGCAAAACCTGAGC	60
PCDH12	AGAACCTGAACCTTCCCGAG	GTTCAGCCGATCACAAAGCT	60
MLL3	TGTCTTCCTCATTGAATTCCTCC	TTGTGAGAACTGGGAGCTCA	60
OR52I1	TCCACTGAGTTGGATGATGAAT	ACTCCATATTCCCTCCAGCA	60
ORC3	GGAAGAGATGCAGTTCTGAGTG	CTGAATTGCCGACTCTCTGC	60
SPTBN2	TGTCTGCTTGTTGGTCCCTA	TGACTTCCTTTTAGCCCTGGT	60
DPEP1	CAATGCATCTCCTCACGTGG	GAAGTTCACCATCACCAGGC	60
DPH1	CGGAGTCACTTCCTAGCTGT	TCGCAGACTTTGGTTAGGGT	60
KCNAB3	GGTTCCCACTTTTCACGTGG	AGACCACAGGCTAGAGGGTA	60
ZNF334	GGGGAGACAGACTGAAAGGA	TTGGCTTCTCTCCTCTGTGA	60
HTR2B	GAAAAGGTGGCAATGCTGGA	CCAAATGCATCCCGAAATGTC	60
FAT2	AGACCTGCCCAAGTCATCAT	GCCTTTCTGTCCTGCAAGTT	60
MRPL2	CCTCCTTACTCCAGTGTGCT	GCAACCAGAGCTATGTCTGC	60
NFE2L3	TTCTGAACCTTTTCCGTGGC	ACATTGTGCTTGCTCTCTCT	60
MATN2	ATCCACCCGCCTTCCAAAG	AGTCCTGAGGTCTCCCATCT	60
LIMS2	GAAGCCCACTCCACAGTCA	CAAGCATGGACAGCAGGAG	60
ARAP3	GTGCTCAGCCTTGCCATTTT	AGCAACACCCAACCCAAATC	60
DOK2	GCCTGGCCAATAACCTGCTT	TCATGCGGCCGAGAGTAG	60
KIAA0513	GTATCCCCACTGCACAGGAC	CCCTTCAGCTTGGTCTCCA	60
YSK4	TCAAGTGTCACCTGAGGCAT	CACAGCCAAAGTCAATCAGCT	60
PARVB	TATTTGGGGTGGGGAGGAAC	CAGATCTATCGGGCTCCTCA	60
FETUB	CTGGGCCTTGTTCTCCACA	TGGCAGAGGAGAACAGAGAG	60
MDH1B	TGGGGATCAAAGACACTTGAG	AAGTCCAGGCAGCTAATGGA	60
CLTCL1	TCCCTCAACCTGTCCATCTG	TGTGCCCTCTGATATCTCTGG	60
IQGAP2	GGTACCCTCTGTGAAGGTGG	TGTCTTCACTACAGGGGCTT	60
ZNHIT1	TGGCAATGGGGATGAGATCA	TCCCACAGCCTGAGTTGG	60
TNC	AGTTGACTTTCAGCCCCAGA	AAGCAGCCCTTTCAAAGTGG	60
C1QTNF9B	CTCCTCTGGGTTTCTGTTTCC	ATGGGTTGTGAGTGGGTAGG	60
TBRG4	GTAGCCCCTTCTCTCTCCT	GCCGGATAAGTACCATTGCTG	60
MYOM2	TGTCACAGCAACGGGACATT	ATTCCCTCCCTTCAGCATCT	60

**Table 3:** Fourteen identified gene mutations in the GEFS+ pedigree were confirmed by Sanger sequencing

Chromosome	GeneName	Substitution	IV−2	III−4	III−5	III−6	III−7	II−5	II−6
chr17	DPH1	V370L	−	＋	−	＋	−	−	＋
chr5	ARAP3	V875A	−	＋	−	＋	−	−	＋
chr2	LIMS2	R343Q	＋	＋	−	−	−	＋	−
chr8	MATN2	R95L	＋	＋	−	−	−	＋	−
Chr7	NFE2L3	R568S	−	＋	−	＋	−	＋	−
Chr9	TNC	E1056K	＋	＋	−	−	−	−	＋
chr8	MYOM2	C1291F	＋	＋	−	−	−	−	＋
Chr2	MDH1B	A377V	−	＋	−	＋	−	−	＋
Chr6	ORC3	R597Q	＋	＋	−	−	−	−	＋
Chr11	OR52I1	G246V	＋	＋	−	−	−	−	＋
Chr13	C1QTNF9B	R42K	−	＋	−	＋	−	−	＋
Chr17	KCNAB3	H258R	＋	＋	−	＋	−	−	＋
Chr4	MANBA	R638H	＋	＋	−	−	−	−	＋
Chr5	PCDH12	R946W	＋	＋	−	−	−	−	＋

The one mutation found in all three affected individuals in this pedigree was the c.773A>G (p.H258R) mutation in the *KCNAB3* gene (OMIM: 604111; Figure 1). Its presence in an unaffected 65‐year‐old family member was probably related to genetic incomplete dominance for GEFS+. The c.773A>G mutation in the *KCNAB3* gene was not found in other unaffected persons of this family or in the 300 unrelated control subjects.

The presence of c.773A>G in the *KCNAB3* gene caused amino acid 258 to change from a histidine residue to an arginine residue (p.H258R). This missense mutation in the *KCNAB3* gene may have affected protein function. The change in amino acides was based on the evaluations by SIFT (prediction score, 0.00) and PROVEAN (prediction score, −3.942).

### 3.3 Functional verification

**Figure 4:**
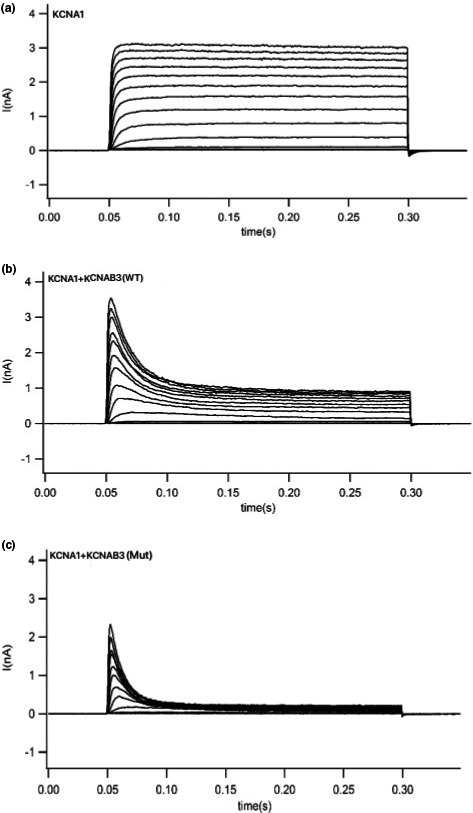
Original current diagram of patch‐clamp experiment (*n* = 11). (a) Potassium current of HEK293 cells transduced with *KCNA1* gene. (b) Potassium current of HEK293 cells transduced with *KCNA1 + KCNAB3*(WT) gene. (c) Potassium current of HEK293 cells transduced with *KCNA1 + KCNAB3*(Mut) gene

We used the patch‐clamp technique to record the cell currents produced by potassium voltage‐gated channel subfamily A member 1 (*KCNA1*), *KCNA1*+ wild‐type *KCNAB3,* and *KCNA1*+ mutant *KCNAB3* (p.H258R mutant type). We found that only the *KCNA1* current was not inactivating. When *KCNAB3* is coexpressed, the current shows obvious deactivation characteristics (Figure 4). This indicates that *KCNAB3* plays a regulatory role in potassium voltage‐gated channels and can promote the inactivation of the potassium current. *KCNAB3* mutation can further accelerate the inactivation of potassium channels and promote the closure of potassium channels. Potassium channel inactivation results in an increase in membrane potential and thus an increase in neuronal excitability, which in some cases may contribute to epilepsy or convulsions.

**Figure 5:**
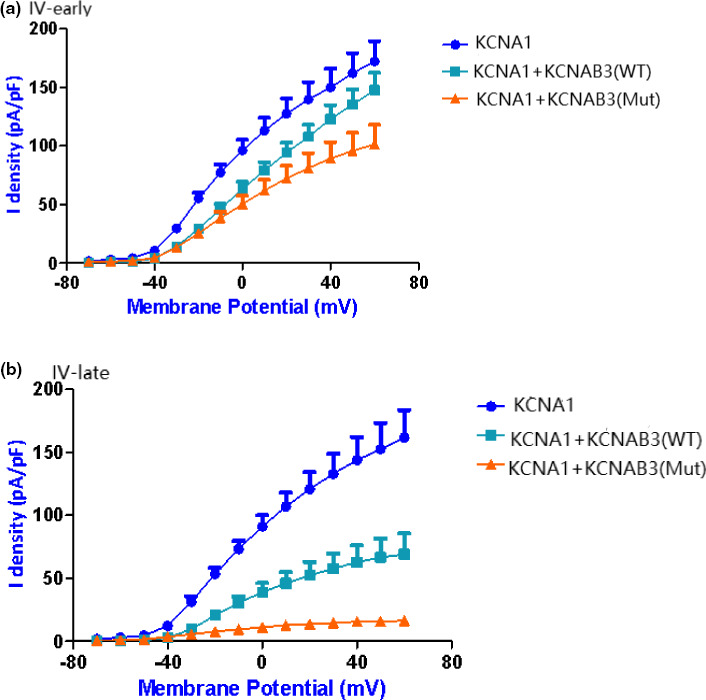
I‐V curve of *KCNA1*. (a) IV‐early curve indicates that wild‐type *KCNAB3* has little influence on the peak current of *KCNA1,* whereas mutant *KCNAB3* can significantly reduce the peak current of *KCNA1*; (b) IV‐late curve indicates that *KCNAB3* mutation caused a significant decrease of the potassium current

We further analyzed the current I–V curve (Figure 5) of *KCNA1*. Because the β subunit is associated with current inactivation, we calculated the difference between the early part and late part of the curve. The early part of the current I–V curve indicates that wild‐type *KCNAB3* has little influence on the peak current of *KCNA1,* whereas mutant *KCNAB3* can significantly reduce the peak current of *KCNA1*. The late part of the curve showed that the *KCNAB3* mutation caused a significant decrease of the potassium current (consistent with the original recording of the cell currents), which suggests that *KCNAB3* can promote inactivation.

## 4 DISCUSSION

In this study, a novel missense mutation, c.773A>G (p.H258R), was found in the *KCNAB3* through the use of whole‐exome sequencing and that this mutation was shared by all three family members with GEFS+ from three generations of an affected family. This mutation was also present in one unaffected individual, which we think may be associated with incomplete penetrance. While it would be helpful to collect further members of the family (III‐1, for instance), this was not done because of death or rejection or unable to contact. It was not found in any of the 300 control subjects. This mutation may lead to changes in protein function in the *KCNAB3* gene, according to results obtained with the bioinformatics tools SIFT and PROVEAN. Functional verification showed that the *KCNAB3* mutation could accelerate the inactivation of potassium channels, thus inhibiting potassium current, increasing neuronal excitability, and promoting epileptic convulsion.

*KCNAB3* consists of 404 amino acids, contains 14 exons on chromosome 17p13.1, and encodes potassium voltage‐gated channel subfamily A regulatory β subunit 3. It belongs to the Shaker family of potassium voltage‐gated channels. The potassium voltage‐gated channel β subunit (Kvβ) is an auxiliary unit (Heinemann et al., 1995) that can stabilize or improve the stability of the potassium voltage‐gated channel α subunit (Kvα) on the cell membrane; furthermore, Kvβ is also a receptor for redox reaction regulation and hypoxia. Kvβ combines with various types of Kvα to form complete neuron type A potassium currents in many regions.

To date, three types of Kvβ have been identified—Kvβ1.1, Kvβ2, and Kvβ3—all of them are highly expressed mostly in the brain. Studies of rat brain tissue have shown that these three subunits are expressed in different regions of the brain: Kvβ1.1 and Kvβ2 are expressed mainly in the hippocampus and dentate gyrus, and Kvβ3 is expressed mainly in the olfactory bulb and thalamus nucleus. In humans (Leicher et al., 1998), messenger RNA of Kvβ3 is expressed specifically in the brain, mainly in the cerebellum.

At present, the functional role of most types of Kvβ is unknown, and the role of *KCNAB3* genes is poorly understood. Kvβ3 has 68% amino acid sequence homology to Kvβ1.1, and the functional role of Kvβ3, as well as of Kvβ1.1, seems to be to introduce inactivation in Kv1.4. In many cases, however, Kvβ3‐induced inactivation, although quite fast, is not complete and can be regulated by the intracellular redox potential (Heinemann et al., 1995). Researchers have reported (Lafrenière & Rouleau, 2012) that the *KCNAB3* gene may be associated with severe migraine, but the mutation site has not been clearly reported; therefore, the relationship between this gene and human neurological diseases needs to be further explored.

In conclusion, a novel missense mutation in *KCNAB3* was found in members of a Chinese Han family who suffered from GEFS+, by means of whole‐exome sequencing. Functional verification showed that *KCNAB3* mutation could accelerate the inactivation of potassium channels, thus inhibiting potassium current, increasing neuronal excitability, and promoting epileptic convulsion. This is the first report about mutation in the *KCNAB3* gene in relation to GEFS+, and our findings may indicate that mutations in the *KCNAB3* gene may be the cause of GEFS+.

## CONFLICT OF INTEREST

The authors declare no conflicts of interest.

## AUTHOR CONTRIBUTIONS

Jian Ding contributed to the conception of the study; Qin‐Fei Miao performed the experiment; Jing‐Wen Zhang and Yu‐Xiong Guo contributed significantly to analysis and manuscript preparation; Yu‐Xin Zhang and Qiong‐Xiang Zhai performed the data analyses and wrote the manuscript; Zhi‐Hong Chen helped perform the analysis with constructive discussions.

### Peer Review

The peer review history for this article is available at https://publons.com/publon/10.1002/brb3.1859.

## Data Availability

The data that support the findings of this study are available on request from the corresponding author. The data are not publicly available due to privacy or ethical restrictions.
